# Improving Early Dementia Detection Among Diverse Older Adults With Cognitive Concerns With the 5-Cog Paradigm: Protocol for a Hybrid Effectiveness-Implementation Clinical Trial

**DOI:** 10.2196/60471

**Published:** 2025-04-03

**Authors:** Rachel Beth Rosansky Chalmer, Emmeline Ayers, Erica F Weiss, Nicole R Fowler, Andrew Telzak, Diana Summanwar, Jessica Zwerling, Cuiling Wang, Huiping Xu, Richard J Holden, Kevin Fiori, Dustin D French, Celeste Nsubayi, Asif Ansari, Paul Dexter, Anna Higbie, Pratibha Yadav, James M Walker, Harrshavasan Congivaram, Dristi Adhikari, Mairim Melecio-Vazquez, Malaz Boustani, Joe Verghese

**Affiliations:** 1 Department of Medicine Montefiore Medical Center/Albert Einstein College of Medicine Bronx, NY United States; 2 Department of Neurology Renaissance School of Medicine Stony Brook University Stony Brook, NY United States; 3 Department of Neurology Montefiore Medical Center/Albert Einstein College of Medicine Bronx, NY United States; 4 Division of General Internal Medicine and Geriatrics Department of Medicine Indiana University School of Medicine Indianapolis, IN United States; 5 Regenstrief Institute, Inc. Indianapolis, IN United States; 6 Department of Family and Social Medicine Montefiore Medical Center/Albert Einstein College of Medicine Bronx, NY United States; 7 Department of Family Medicine Indiana University School of Medicine Indianapolis, IN United States; 8 Department of Epidemiology & Population Health Montefiore Medical Center/Albert Einstein College of Medicine Bronx, NY United States; 9 Department of Biostatistics and Health Data Science Indiana University School of Medicine Indianapolis, IN United States; 10 Department of Health & Wellness Design School of Public Health Indiana University Bloomington, IN United States; 11 Division of Community and Population Health Department of Pediatrics Montefiore Medical Center/Albert Einstein College of Medicine Bronx, NY United States; 12 Departments of Ophthalmology and Medical Social Sciences Feinberg School of Medicine Northwestern University Chicago, IL United States

**Keywords:** cognitive assessment, cognitive screening, cognitive impairment, mild cognitive impairment, dementia, dissemination and implementation science, clinical trial protocol, randomized controlled trial, hybrid implementation-effectiveness trial

## Abstract

**Background:**

The 5-Cog paradigm is a 5-minute brief cognitive assessment coupled with a clinical decision support tool designed to improve clinicians’ early detection of cognitive impairment, including dementia, in their diverse older primary care patients. The 5-Cog battery uses picture- and symbol-based assessments and a questionnaire. It is low cost, simple, minimizes literacy bias, and is culturally fair. The decision support component of the paradigm helps nudge appropriate care provider response to an abnormal 5-Cog battery.

**Objective:**

The objective of our study is to evaluate the effectiveness, implementation, and cost of the 5-Cog paradigm.

**Methods:**

We will enroll 6600 older patients with cognitive concerns from 22 primary care clinics in the Bronx, New York, and in multiple locations in Indiana for this hybrid type 1 effectiveness-implementation trial. We will analyze the effectiveness of the 5-Cog paradigm to increase the rate of new diagnoses of mild cognitive impairment syndrome or dementia using a pragmatic, cluster randomized clinical trial design. The secondary outcome is the ordering of new tests, treatments, and referrals for cognitive indications within 90 days after the study visit. The 5-Cog’s decision support component will be deployed as an electronic medical record feature. We will analyze the 5-Cog’s implementation process, context, and outcomes through the Consolidated Framework for Implementation Research using a mixed methods design (surveys and interviews). The study will also examine cost-effectiveness from societal and payer (Medicare) perspectives by estimating the cost per additional dementia diagnosis.

**Results:**

The study is funded by the National Institute of Neurological Disorders and Stroke of the National Institutes of Health (2U01NS105565). The protocol was approved by the Albert Einstein College of Medicine Institutional Review Board in September 2022. A validation study was completed to select cut scores for the 5-Cog battery. Among the 76 patients enrolled, the resulting clinical diagnoses were as follows: dementia in 32 (42%); mild cognitive impairment in 28 (37%); subjective cognitive concerns without objective cognitive impairment in 12 (16%); no cognitive diagnosis assigned in 2 (3%). The mean scores were Picture-Based Memory Impairment Screen 5.8 (SD 2.7), Symbol Match 27.2 (SD 18.2), and Subjective Motoric Cognitive Risk 2.4 (SD 1.7). The cut scores for an abnormal or positive result on the 5-Cog components were as follows: Picture-Based Memory Impairment Screen ≤6 (range 0-8), Symbol Match ≤25 (range 0-65), and Subjective Motoric Cognitive Risk >5 (range 0-7). As of December 2024, a total of 12 clinics had completed the onboarding processes, and 2369 patients had been enrolled.

**Conclusions:**

The findings of this study will facilitate the rapid adaptation and dissemination of this effective and practical clinical tool across diverse primary care clinical settings.

**Trial Registration:**

ClinicalTrials.gov NCT05515224; https://www.clinicaltrials.gov/study/NCT05515224

**International Registered Report Identifier (IRRID):**

DERR1-10.2196/60471

## Introduction

### Background and Rationale

#### Problem and Progress

Alzheimer disease and related dementias (ADRD) affect approximately 57 million people globally, a figure projected to rise to 152 million by 2050 [1,2]. Studies have long noted deficiencies and delays in individuals receiving a dementia diagnosis [3-12]. The World Health Organization’s Global Action Plan on Dementia includes a focus on the right to a timely dementia diagnosis to enable better planning, treatment, care, support, and quality of life [13]; for example, timely diagnosis can help individuals avoid preventable accidents and injuries leading to care escalation and can reduce distress for patients and caregivers [9,14-16]. In addition, several studies have estimated a potential cost saving of approximately US $10,000 per timely diagnosis because of delayed institutionalization, with the expectation that improved health and quality of life related to early diagnosis could result in even more cost savings [17].

Studies have repeatedly noted that delay and deficiency in diagnosis disproportionately impacts individuals from historically minoritized racial and ethnic groups as well as those from socioeconomically disadvantaged backgrounds [3-12]; for example, a recent nationally representative study found that a higher proportion of non-Hispanic Black and Hispanic individuals had a missed or delayed clinical dementia diagnosis compared to non-Hispanic White individuals (46% and 54% vs 41%; *P*<.001). This is thought to be an underestimate of the impact because the study relied on claims-based data, and non-Hispanic Black and Hispanic individuals may be less likely to access care that generates claims [18].

Experts recommend robust, multifaceted strategies to close these diagnosis gaps [15,17,19-24]; interventions must address implementation (“the actively planned process of putting evidence to use or integrating new interventions within a specific setting.” [25]) and dissemination (“the targeted distribution of information and intervention materials to a specific public health or clinical practice audience” [26]) challenges as much as they aim to achieve high clinical accuracy and quality [20,21,27-31]. The Consortium for Detecting Cognitive Impairment, Including Dementia, a collaborative research effort directed and funded by the National Institute of Neurological Disorders and Stroke and the National Institute on Aging of the National Institutes of Health (NIH), has produced promising paradigms to meet this need. As part of this effort, our team at the Albert Einstein College of Medicine and Montefiore Medical Center (hereinafter Montefiore Einstein clinics) in the Bronx, New York, United States, developed the 5-Cog paradigm [32,33]. In a randomized controlled trial (RCT) of 1200 older adults with cognitive concerns at an urban primary care clinic in a primarily Black and Hispanic community, we showed that the 5-Cog paradigm improves dementia care, most notably by increasing the rate of diagnosis of mild cognitive impairment (MCI) and dementia [33]. This detection and diagnosis increases opportunity for primary care providers (PCPs), patients, and patients’ families to intervene to potentially slow or prevent progression to dementia or, for individuals who do progress, to prepare in advance to meet the complex caregiving and clinical challenges to come [34].

Hence, our research questions for this cluster randomized trial were as follows:

- Can a brief cognitive assessment paired with a clinical decision tree (5-Cog paradigm) increase the rate of new diagnoses of MCI syndrome or dementia in primary care patients presenting with cognitive concerns in real-world settings, across broad patient groups (hence its categorization as a pragmatic trial [35])?
- What are the determinants of the 5-Cog’s impact in these settings?

#### Updates to the 5-Cog Paradigm for This Trial

The 5-Cog paradigm (brief cognitive assessment battery and decision support tool) worked well in our initial trial. However, building on implementation reflections from that trial [27] as well as feedback from local primary care stakeholders, we have made 2 refinements to the 5-Cog paradigm to improve clinical utility and aid future dissemination. We substituted the gait speed measurement component of the 5-Cog with a questionnaire that assesses mobility and cognition. In addition, we modified the 5-Cog decision support component to capitalize on electronic medical record (EMR) capabilities and better align the 5-Cog paradigm with existing care provider workflows. All these modifications are described in detail in the following subsection (The 5-Cog Paradigm) and in the Interventions section.

#### The 5-Cog Paradigm

The 5-Cog paradigm is composed of a 5-minute brief cognitive assessment (the 5-Cog battery) combined with an EMR-embedded clinical decision support tool. The 5-Cog battery was granted US copyright registration effective December 14, 2023.

Clinical decision support provides timely information, usually at the point of care, to inform and improve decisions about a patient’s care [36].

The first of the 3 items in the 5-Cog battery is the Picture-Based Memory Impairment Screen (PMIS), created by Verghese et al [37], which uses 4 pictures to test free and cued recall after a delay of at least 2 minutes. Administration and scoring have been previously described [38]. This screener was selected because of ease of use (simple to administer after minimal training and does not require specialized technology), freedom from literacy bias (uses photographs of items instead of words), and cultural fairness (photographs were chosen and validated for consistent recognition by patients in the relevant cultural milieu). In our validation study, PMIS was shown to have high validity for distinguishing older adults with cognitive impairment from those without, regardless of age, sex, education, or presence of depression (sensitivity and specificity >90%) as well as excellent reliability (intraclass correlation 0.91) [37,38].

The second item in the 5-Cog battery is the Symbol Match [39], developed by one of our coinvestigators (EFW). It is an oral timed transcription task created to identify difficulties with divided attention, visual scanning, tracking, and motor speed. It requires individuals to quickly substitute (verbalize) numbers for an array of symbols using a key provided at the top of the page. After a practice run with 7 symbols, individuals are given 90 seconds to correctly name as many items as they can as quickly as they can without making a mistake. The number of correct oral substitutions at the end of 90 seconds is the participant’s score. Individuals who complete the task before the time limit receive a ceiling score of 65. Internal validation within our study population suggested 25 as the optimal cutoff score to identify cognitive impairment (refer to the Results section for details). The Symbol Match was chosen because of ease of use (requires no specialized equipment and minimal training to administer) [39]. The 90-second Symbol Match correlates highly with the established Symbol Digit Modalities Task [40,41], which can detect nonmemory impairments and functional changes [42]. This feature allows Symbol Match to detect patients with nonmemory impairments that may not be identified by the PMIS [39].

Finally, the 5-Cog incorporates an assessment of gait. The motoric cognitive risk (MCR) syndrome, first described by Verghese et al [43], and defined as slow gait in the presence of cognitive concerns, has been extensively validated to predict an elevated risk of dementia independent of other cognitive assessments [43-47]. Traditionally, the MCR syndrome assessment involves the measurement of gait speed. Given that gait speed is not routinely tested in current primary care environments and that it may present a barrier for 5-Cog implementation, we modified this assessment. For this study, we will be assessing the Subjective MCR (sMCR) screening tool, developed by Ayers et al [48], which uses the patient’s subjective reports of mobility and cognitive concerns via a 5-item questionnaire, rather than having a tester measure gait speed. Responses to these 5 questions are used to derive a weighted score that is used to define sMCR. Administration and scoring have been previously described [48]. We found the sMCR approach to have excellent discriminative validity versus objective MCR (defined using gait speed) and excellent predictive validity for incident dementia; in our validation study, participants who met the sMCR criteria had a >2-fold increased risk of developing dementia [48].

The 5-Cog paradigm’s EMR-based decision support tool includes 2 components: documentation of the patient’s 5-Cog battery result and recommended next steps that may be appropriate for further diagnosis or management of the patient’s cognitive concerns (clinical decision support). This “nudge” supports and supplements the provider’s own clinical decision–making process [49,50]. In this study, the decision support tool incorporates 3 additional features that make it resemble other EMR-based decision support systems: first, the 5-Cog result will be listed among other clinical practice advisories that contain information tailored to each patient regarding time-sensitive care needs. Second, providers will receive 5-Cog results through an interruptive alert when they access the patient’s chart (as they do in cases of other clinical issues that can impact patient safety). Finally, the decision support tool will include a direct link to orders for the next steps in care. This reduces clinician cognitive load by providing a menu of options (such as appropriate laboratory, imaging, and referral orders) and minimizes clicks by consolidating these options in one place.

**Figure 1:**
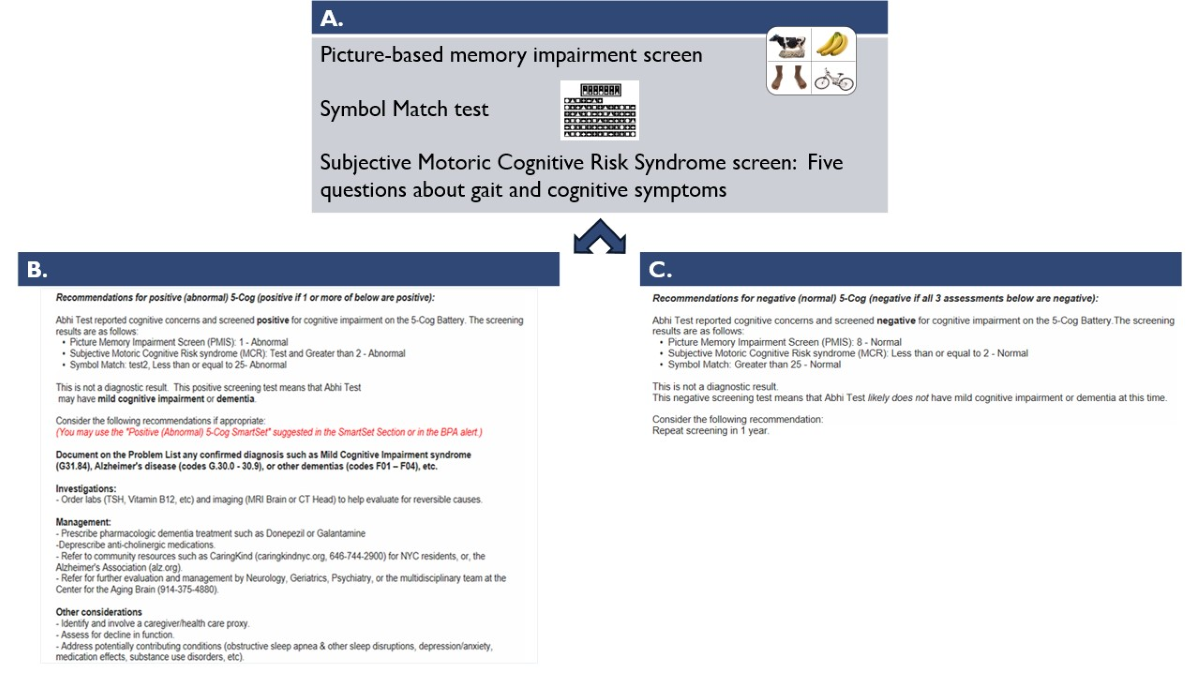
(A) The 5-Cog battery (5-min cognitive assessment). (B) Decision support for a patient with a positive 5-Cog result. (C) Decision support for a patient with a negative 5-Cog result. EMR: electronic medical record.

Our 5-Cog 1.0 protocol [32] can be referenced for additional detail on the development and design of the 5-Cog paradigm. Figure 1 depicts the 2 components of the 5-Cog paradigm.

#### Implementation Study

The United States Institute of Medicine (now the National Academy of Medicine) estimated a 17-year gap from when a clinical innovation is proven effective to when it is routinely implemented in clinical care [51]. Given that cognitive impairment detection paradigms are not adopted widely in primary care, it is important to systematically identify and address potential factors that influence implementation outcomes, that is, implementation determinants [52]. Our implementation measurements for the 5-Cog will be guided by the Consolidated Framework for Implementation Research (CFIR). The CFIR is an established implementation science theoretical framework for identifying implementation determinants [53,54]. We chose the CFIR because it facilitates the identification of implementation determinants that influence implementation outcomes either positively as facilitators or negatively as barriers [55-58]. Each CFIR construct is well defined, has established measures, and can be depicted in rich qualitative detail. The CFIR is practical because it is often used to both explore and subsequently optimize future implementation contexts and processes [59]. As with many implementation science frameworks, the CFIR does not mandate a specific data collection methodology [53]; studies applying the CFIR have used quantitative only, qualitative only, or mixed methods approaches [59]. We have chosen a mixed methods strategy to examine implementation contexts, processes, and outcomes. The study approach is based on the broader epistemology of critical realism [60] and more specifically the realist evaluation framework [61] wherein quantitatively measured implementation outcomes are explained by analyzing the relationships between implementation context and process factors. We will use the CFIR to identify implementation determinants through qualitative processes (interviews). We will use quantitative measures—the Acceptability of Intervention Measure (AIM), the Feasibility of Intervention Measure (FIM), and the Intervention Appropriateness Measure (IAM) [62]—to measure implementation outcomes. We will then analyze all findings using the CFIR as a lens to examine the relationships between these outcomes and implementation context and process factors [54,59].

#### Cost-Effectiveness Assessment

Currently, Medicare reimburses cognitive impairment assessments for older adults as part of their annual wellness visit (AWV) [22]. However, there is no standardized required form and output for this assessment. For health systems that would like to implement the 5-Cog for the AWV, questions may arise regarding its impact on costs. The 5-Cog battery is a low-cost intervention, requiring simple paper-and-pencil tools that are easily adapted to digital formats and minimal training to administer. For the 5-Cog decision support component, we anticipate programming and workflows to be translatable between EMR systems with a 1-time, upfront customization and without recurring software or staffing costs. Thus, the 5-Cog’s direct expenses predominantly include staffing costs (eg, salaries, benefits, and travel expenses for workers administering the 5-Cog). Given the short duration and minimal supplies cost of the 5-Cog, indirect expenses (eg, scheduling, record keeping, facility overhead, and equipment depreciation) and opportunity costs (eg, forgoing other billable care) are anticipated to be minimal. Investigators have previously reported costs associated with both community health workers (CHWs) and practice facilitators throughout Indiana and the Midwest in the United States [63]. There may also be costs associated with dementia care actions resulting from the use of the 5-Cog. However, these follow-up costs may be offset by cost savings resulting from earlier dementia care intervention [64].

The goal of our budget impact analysis (BIA) [65-67] and cost-effectiveness analysis (CEA) [68] is to understand the cost per cognitive case identified from a societal and payer (eg, Medicare) perspective to facilitate informed dissemination (per the CFIR model). The study design for estimating the financial effects involves addressing several issues, many predetermined by BIA and CEA best practices and the Consolidated Health Economic Evaluation Reporting Standards [65-68]. The BIA will determine financial planning and affordability, and the CEA will determine the effectiveness and value of the 5-Cog compared to enhanced usual care. The BIA can be used to determine financial adoption and scalability for national implementation.

### Trial Design

Given the problems of underdiagnosis and undertreatment of dementia, which are expected to worsen with the growing number of aging adults globally [69], we chose a hybrid type 1 effectiveness-implementation design for this trial. In this design, the primary emphasis is on testing the intervention, with a secondary emphasis on exploring implementation-related factors [55]. We chose this design because it would be premature to conduct an implementation-only trial, given that the 5-Cog has previously been studied in 1200 individuals in only 1 urban primary care setting. The hybrid type 1 effectiveness-implementation design allows us to confirm the effectiveness of our intervention, while also hastening its potential to make needed impact in the “real world” through incorporating implementation examination prospectively [55]. The clinical effectiveness (in this case, meaning the degree of beneficial effect [70]) component of the trial will be completed using a pragmatic, cluster randomized design (randomized at the level of the clinic) with intervention clinics receiving the 5-Cog paradigm and control clinics receiving enhanced usual care (cognitive concern information and cognitive impairment detection education provided but no detection approach or clinical decision support tool implemented).

### Objectives

This trial’s three aims are to (1) test whether the 5-Cog paradigm used in primary care patients with cognitive concerns in diverse urban and rural environments and including individuals from racial and ethnic minority groups and those with socioeconomically disadvantaged backgrounds leads to an increased rate of MCI and dementia diagnoses; (2) explore the implementation context, process, and outcomes of the 5-Cog paradigm in diverse primary care clinics using mixed methods guided by the CFIR; and (3) assess the cost-effectiveness of the 5-Cog paradigm.

We hypothesize that this trial will demonstrate the 5-Cog paradigm’s effectiveness in increasing the diagnosis of MCI in dementia and primary care, its cost-effectiveness in achieving this outcome, and the range of factors shaping its successful implementation across diverse primary care settings.

## Methods

### Participants, Interventions, and Outcomes

#### Study Setting

The study is being carried out at primary care clinics affiliated with 2 large academic health systems: Montefiore Medical Center and Albert Einstein College of Medicine (hereafter referred to as Montefiore-Einstein) in the Bronx, New York, and Indiana University Health (IUH) in Indiana.

The Montefiore-Einstein clinics serve an urban population primarily composed of individuals from historically minoritized racial and ethnic groups (Hispanic/Latino and Black populations). Of note, these clinics serve Bronx County, which ranks last (62 out of 62) among New York State counties on health-related indicators [71] and has a significant poverty rate, affecting 1 in 4 older adults [72]. The IUH clinics are situated across both urban and rural settings. Within this network, 12 (75%) of the 16 selected clinics are located in regions scoring >60 on the national area deprivation index (score range 0-100; higher scores indicate higher deprivation) [73,74]. Notably, 2 (17%) of these 12 clinics are in areas with an area deprivation index score of >91, underscoring the profound level of need of these communities. Indiana health-related indicators show higher premature death, lower life expectancy, lower PCP and mental health provider availability, and lower median household income than the US average [74].

The study procedures are completed by research assistants (RAs), who are trained as CHWs and embedded in the clinics. This approach was chosen for its pragmatic value, reflecting reality in primary care settings in which CHWs are incorporated into clinical teams and may complete other screening questionnaires with patients [75,76] and because the 5-Cog components have been shown to be practical and feasible for administration by individuals who are not medical professionals and with minimal training [37,44,77]. The RAs at Montefiore-Einstein are bilingual in English and Spanish because of the high proportion of Spanish-speaking participants at this site. All study materials were translated from English into Spanish and back translated to ensure language fidelity.

#### Eligibility and Screening

Clinics are eligible to participate if they provide primary care (internal medicine or family medicine), not specialty care. Clinics primarily serving as residency teaching sites are excluded.

In keeping with the pragmatic nature of the trial, our individual-level eligibility criteria are designed to ensure that enrolled patients are those who would receive this intervention if it were part of usual care [78-80]. The 5-Cog intervention was designed to improve dementia diagnosis in ambulatory primary care settings. Individuals are excluded if they have a prior diagnosis of dementia or reside in a nursing home. Nursing home residents are excluded because they already have a high prevalence of diagnosed dementia (60%-90%) [81] and largely do not receive primary care from ambulatory clinics because of their mobility and cognitive limitations [82]. To be eligible for our 5-Cog effectiveness study, individuals must be aged ≥65 years, have no prior diagnosis of dementia, speak English (Montefiore-Einstein or IUH) or Spanish (Montefiore-Einstein only), have a scheduled primary care office visit, and endorse a cognitive concern via a subjective cognitive concern questionnaire (SCQ) [32,83]. Initial screening is completed by RAs via prospective EMR review of patients scheduled at each clinic and confirmed through screening telephone calls.

Implementation study participants will be recruited from among patients, caregivers, clinicians, clinic staff, clinical leaders, clinical informatics staff, and administrative leaders. The exclusion criteria are the same as those for the effectiveness study.

Textbox 1 lists the complete individual-level inclusion and exclusion criteria, including the SCQ.

**Inclusion and exclusion criteria.**

**Effectiveness study**

- Individual-level inclusion criteria
  Aged ≥65 years
  English or Spanish speaking (Albert Einstein College of Medicine and Montefiore Medical Center will enroll English- and Spanish-speaking patients; Indiana University Health will enroll only English-speaking patients)
  Have a cognitive concern: subjective cognitive concern questionnaire result is positive (≥1 of the following: “Are you concerned about your memory?”—the chosen response is yes; “Are your loved ones concerned about your memory?”—the chosen response is yes; and “Is your mind as clear as it used to be?”—the chosen response is no)
- Individual-level exclusion criteria
  Prior diagnosis of dementia (as recorded in the electronic medical record or reported by primary care provider; the prior diagnosis of mild cognitive impairment is not an exclusion criterion, but the effectiveness outcome is only counted if a participant previously diagnosed with mild cognitive impairment is assigned a new diagnosis of dementia)
  Nursing facility resident

**Implementation study**

- Individual-level inclusion criteria
  - Patients who have undergone the 5-Cog battery, caregivers of these patients, clinicians, clinic staff, clinical leaders, clinical informatics staff, and administrative leaders at each of the randomized primary care practices
  - Ability to provide informed consent
- Individual-level exclusion criteria
  Prior diagnosis of dementia (as recorded in the electronic medical record or reported by primary care provider; the prior diagnosis of mild cognitive impairment is not an exclusion)
  Nursing facility resident

#### Recruitment of Clinics and Patients

The study team actively recruited clinics in the Bronx and Indiana to participate through contacting their administrative leadership to discuss the study. Generally, 1 to 3 meetings were held with clinic leadership and practicing clinicians to introduce the study and secure the clinic’s commitment to participate.

All individual patient recruitment for the effectiveness study is passive. RAs review a clinic’s scheduled patients via the EMR to determine which patients seem to meet the eligibility criteria and contact potentially eligible patients by telephone within 1 week before their scheduled office visit. The patient is informed that the caller is working with the patient’s primary care practice to assess cognitive concerns. The patient is asked to confirm that they plan to keep their upcoming clinic appointment. If they have decided to change the appointment, cognitive concerns are not assessed. If the patient confirms their scheduled appointment, the RA administers the SCQ (Textbox 1). The patient is free to decline to answer, at which point the call is ended. When the patient answers the questions, the results are documented in the EMR. In the clinics randomized to the control arm, patients who have answered the SCQ are considered recruited; and if their SCQ result is positive, they are considered enrolled once they show up to their scheduled clinic appointment. In the clinics randomized to the intervention arm, patients with a positive cognitive concern are invited to undergo additional cognitive screening (the 5-Cog battery) at the clinic on the day of their office visit, just before they see their care provider. Patients who agree are considered recruited, with enrollment confirmed once they complete their scheduled office visit. In both arms, patients who do not show up to their scheduled clinic appointment after a positive SCQ result are not considered enrolled. If these patients have another scheduled clinic appointment during the study period at their clinic, they are eligible to be rerecruited and enrolled at this time (RAs will call and confirm SCQ responses before this visit).

Implementation study participants will be actively recruited from intervention sites through snowball sampling among different types of key informants mentioned previously. In addition, we will collect quantitative implementation surveys from as many clinicians and staff as possible at the intervention clinics.

#### Participant Timeline

The 22 participating clinics were randomized in year 1 of the study. These clinics are onboarded in waves throughout the 5-year study period. The time period over which each clinic will experience active study recruitment (with the goal of enrolling 300 patients per clinic) is anticipated to vary from 3 to 12 months, depending on clinic size.

For individual patients in the effectiveness study, the initial screening and recruitment telephone call takes place within 1 week before a patient’s scheduled primary care visit and takes 10 to 15 minutes. Patients are then considered enrolled when they complete their scheduled PCP clinic appointment.

At the intervention clinics, participating patients are asked to arrive up to 1 hour early for their scheduled visit to allow sufficient time for undergoing the 5-Cog battery before meeting their care provider. The 5-minute assessment is completed at the clinic before the appointment.

In both intervention and control groups, patients remain enrolled for 90 days. Study outcomes are ascertained at 90 days (primary and secondary end points), at which point the enrolled patients are considered to have completed participation. This time window was selected because >95% of physician actions for dementia care were completed within this period in our initial trial [33].

**Figure 2:**
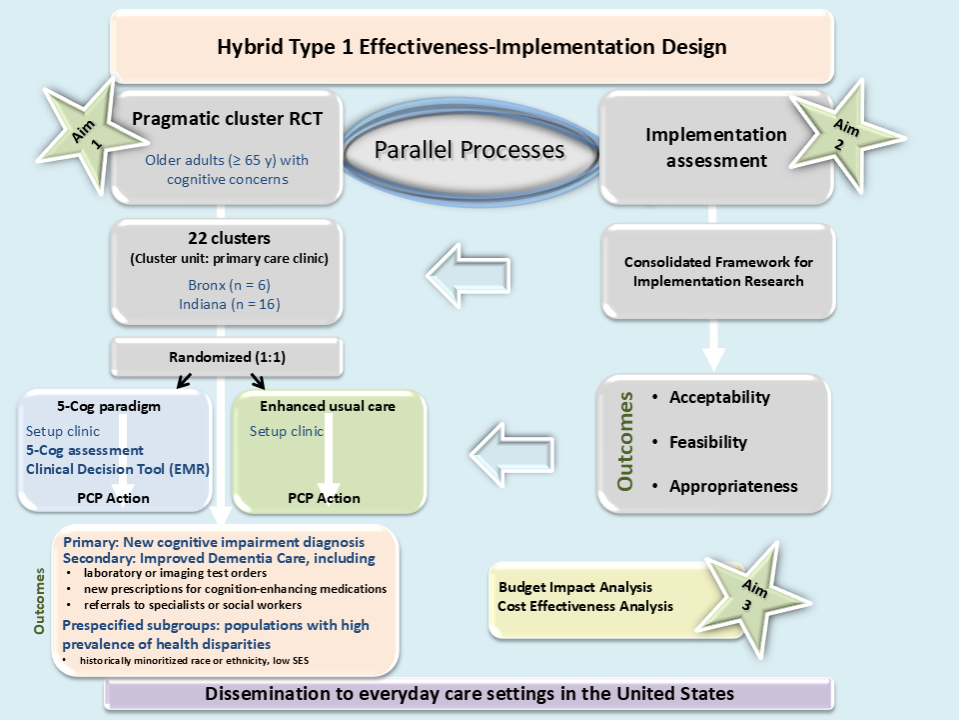
Study flow. EMR: electronic medical record; PCP: primary care provider; RCT: randomized controlled trial; SES: socioeconomic status.

Implementation study participants are enrolled when they consent to participate in a survey or an interview, and their enrollment is completed at its conclusion. Figure 2 depicts the study flow.

#### Planned Sample Size

The planned effectiveness study sample size is 6600 enrolled patients, including approximately 300 (4.56%) from each of the 22 participating clinics (Bronx: n=6, 27%; Indiana: n=16, 73%).

For the implementation interviews, we will use snowball sampling to identify the most relevant informants across clinics of different sizes. We anticipate conducting up to 6 implementation interviews per practice for a maximum total sample size of 132 participants or until thematic saturation is reached over the 5-year study period. We will disseminate the quantitative implementation surveys to the clinicians and staff at the intervention clinics.

#### Interventions in the Intervention Arm (5-Cog Battery With Decision Support Tool)

On the day of the patient’s scheduled clinic visit, an RA meets the patient upon their entry into the clinic waiting area in preparation to complete the 5-Cog with them before their PCP visit. The RA escorts the patient through clinic registration. At the same time, the RA communicates with the clinical team to let them know that the patient will undergo the 5-Cog battery before being ready to see their care provider.

The RA administers the 5-Cog (refer to the Introduction section and Figure 1 for more details on the 5-Cog) in a private space within the clinic and then brings the patient back to the waiting area. Next, the RA informs the clinical team that the patient has completed the 5-Cog battery and is ready to be integrated back into the usual clinical workflow. The RA then completes documentation of the battery results in the EMR (and in the study database). The 5-Cog decision support recommendations are harmonized across sites for a positive 5-Cog result, as they are for a negative 5-Cog result (Figure 1).

Montefiore-Einstein and IUH use 2 different EMR systems: Epic (version 100.2412.0.0) and Cerner (version 2024.3), respectively. Unique features within each EMR are used to capture and house the 5-Cog results, create alerts, and present the results and decision support recommendations to the care providers.

At Montefiore-Einstein, the RA creates a “research note.” Here, they input the 5-Cog battery component scores by selecting from a drop-down list for each assessment. They also manually select the appropriate decision support arm (for positive vs negative 5-Cog result) using a quick-text feature. The EMR then responds to the results of the 5-Cog assessments, using structured data elements. Thus, the values that the RA enters for the three 5-Cog battery components trigger a preprogrammed tailored care provider alert, a “best practice advisory” (BPA). If the result of any of the 3 assessments indicates potential cognitive impairment, a “positive 5-Cog” BPA is triggered. If the results do not indicate potential cognitive impairment a “negative 5-Cog” BPA gets triggered. The BPA is “interruptive”; as soon as the care provider accesses the patient’s medical record for the current visit in the EMR, they are shown the alert on their screen. The BPA contains the 5-Cog result with a hyperlink to the research note.

At IUH, the RA inputs the 5-Cog battery result into an EMR-based flowsheet through ad hoc documentation. Specifically, these results are entered into the “Results Review - Clinical Assessment” tab. The EMR is programmed to automatically trigger the appropriate decision support content based on the 5-Cog battery result. As at Montefiore-Einstein, a customized, interruptive alert is displayed to the provider. At IUH, the alert itself contains the 5-Cog result, along with all decision support recommendations.

In both IUH and Montefiore-Einstein EMR flows, care providers must respond to the interruptive alert, acknowledging it in some way. The options for this vary by EMR. At both sites, the alert for patients with a positive 5-Cog result also facilitates linking to an order set. This allows the care provider to efficiently and comprehensively select clinical actions that may be relevant to the patient’s further cognitive care.

**Figure 3:**
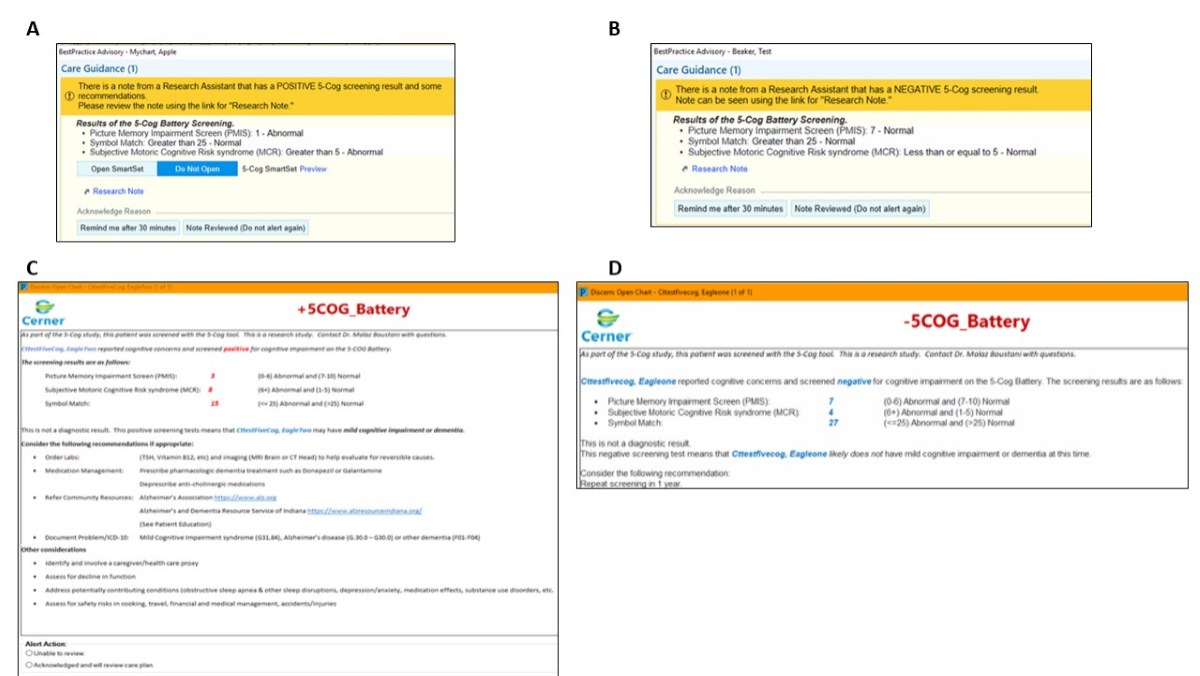
Electronic medical record (EMR)–based provider alerts for the intervention sites at the Albert Einstein College of Medicine and Montefiore Medical Center (Montefiore-Einstein; Epic EMR system) and Indiana University Health (IUH; Cerner EMR system). (A) Montefiore-Einstein: positive 5-Cog result. (B) Montefiore-Einstein: negative 5-Cog result. (C) IUH: positive 5-Cog result. (D) IUH: negative 5-Cog result.

Figure 3 depicts a sample of the Montefiore-Einstein and IUH EMR alerts.

**Figure 4:**
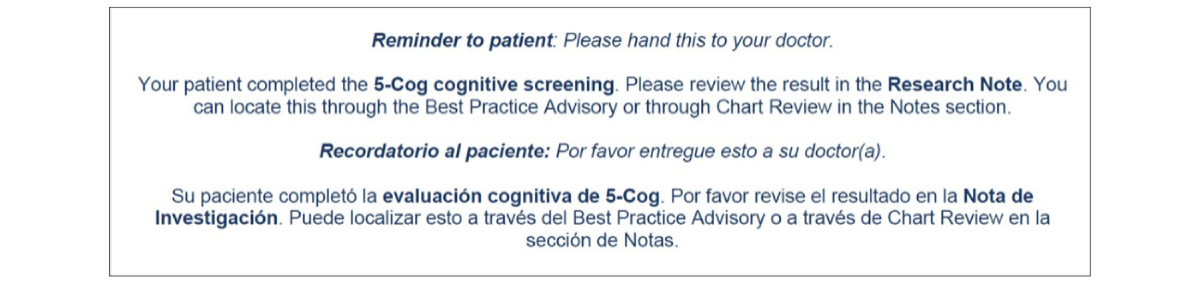
Token to alert care provider for patient's 5-Cog participation.

At both sites, a paper token (Figure 4) is used as an additional feature to help ensure that care providers review the patients’ 5-Cog results. Patients are handed this token after they complete the 5-Cog battery and are asked to hand it to their care provider at their scheduled visit, generally within 30 minutes after the 5-Cog battery administration.

#### Interventions in the Control Arm (Enhanced Usual Care)

**Figure 5:**
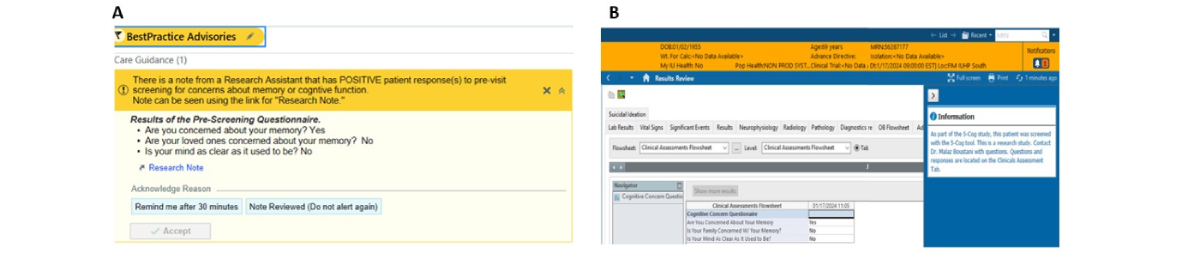
Electronic medical record (EMR)–based care provider alerts for the control sites at the (A) Albert Einstein College of Medicine and Montefiore Medical Center (Epic EMR system) and (B) Indiana University Health (Cerner EMR system).

Patients are screened via telephone using the SCQ, as described in the Recruitment of Clinics and Patients subsection. RAs input results into the EMR. Care providers receive positive SCQ results through noninterruptive alerts. Figure 5 presents images of the alerts at Montefiore-Einstein and at IUH. We are calling this arm “enhanced usual care” because care provider are informed about the presence of a cognitive concern in their patient in this group. In addition, as at the intervention sites, care providers at the enhanced usual care sites receive preparatory education (described in the next subsection). The care providers at the control and intervention sites do not overlap.

#### Care Provider Education and Preparation in Both Arms

Care providers at both control and intervention sites receive Microsoft PowerPoint presentations on the 5-Cog study. The presentations review the background to the study and then explain the study interventions that will be carried out at the sites (SCQ and new EMR features at both sites; 5-Cog at intervention sites). The presentations last 10 to 15 minutes at the control sites, and 25 to 30 minutes (to allow education about the 5-Cog battery) at the intervention sites. These presentations are provided 2 to 4 weeks before study start at a regularly scheduled clinic staff meeting. In addition to this education, care providers are given brochures that review cognitive impairment detection, diagnosis, treatment, and billing and invited to contact study staff at any time with questions. Finally, they are given tip sheets summarizing the new EMR features. At the intervention sites, care providers are updated on study progress via a monthly email or an informal in-person check-in by a study leader.

Of note, at both intervention and control sites, members of the interprofessional clinic leadership team (administrative and nursing managers) are provided with education on the study procedures and flow so that they can be prepared to assist and appropriately direct any patients who have questions about the study. At the intervention sites, members of the interprofessional clinic leadership team are also engaged in a session to plan clinic-customized logistical workflows to minimize interruptions to the clinic’s operations during the course of the study. These workflows, along with a background to the study, are presented to the full interprofessional clinic staff before study initiation.

#### Interventions for the Implementation Study

From the intervention sites, a subset of patients (after the completion of study procedures and clinic visit) and providers will be invited to complete interviews to assess implementation issues. We will adapt the semistructured CFIR interview question guide. Interviews will be conducted, recorded, and analyzed by study staff trained in qualitative interviewing. We will aim to include patients with both positive and negative 5-Cog results. PCPs and clinical staff at the intervention sites will be asked to complete quantitative surveys containing 3 standardized 4-item measures: the AIM, FIM, and IAM [62]. The implementation outcomes measures are written at a fifth grade reading level; therefore, no specialized training is needed for administration, scoring, or interpretation. Finally, we will retain and later analyze recordings and field notes from our preimplementation stages (initial conversations with clinics and readiness presentations) through the completion of the project. This analysis will assess the barriers and facilitators to primary care sites’ buy-in for study engagement. We will also use summary reports to discern trends in care provider responses to the EMR-based alerts.

#### Interventions for the Cost-Effectiveness Study

Billing and actuarial data will be extracted from the EMR for cost estimation. In parallel fashion to the intervention sites, costs are also collected at the control sites. Standardized cost calculators previously developed by investigators [67,84] will also be used to facilitate microcosting (measuring detailed resource use and unit costs to obtain precise estimates [85]) applicable to the 5-Cog paradigm. We will evaluate the cost of implementing and sustaining the 5-Cog paradigm by accounting for and itemizing specific program characteristics. We will evaluate differences in 5-Cog costs of care on the desired study outcomes compared to enhanced usual care.

#### Effectiveness Study: Primary Outcomes

The primary outcome is a new diagnosis of MCI or dementia within 90 days after study enrollment. Patients who enter the study with a known diagnosis of MCI will only be counted as meeting the primary outcome if they receive a new diagnosis of dementia. We chose diagnosis as the primary outcome for 2 reasons. First, a new diagnosis was a common outcome in our 5-Cog 1.0 study, and we want to validate this finding [33]. Second, this outcome is highly relevant to patients and PCPs: knowing their cognitive diagnosis has been shown to be important to patients and family members [34,86,87]; moreover, diagnostic clarity facilitates appropriate psychoeducation, resource connections, tailored medical treatment, and caregiver activation [88,89].

#### Effectiveness Study: Secondary Outcomes

The secondary outcome is a composite: “improved dementia care” within 90 days after study enrollment. “Improved dementia care” is defined by any of the following actions that are relevant to dementia diagnostic assessment and care: orders for dementia-related laboratory or imaging tests; a new prescription for cognition-enhancing medication or the deprescribing of anticholinergic medication; or referrals to a dementia specialist clinician, a nurse navigator, a social worker, or a community-based organization for related supports. We are including this composite outcome because a new diagnosis without subsequent action is less likely to improve patient outcomes. The aforementioned components are highly relevant to both PCPs and patients [90,91]. The actions described are only counted as an outcome if a cognitive indication (eg, memory loss or MCI) is entered as the indication in the EMR. Tests or referrals without indications entered or performed for any other medical reasons are not counted as outcomes. Prescription of dementia medications will always be counted because these medications inherently indicate cognitive impairment. For anticholinergic deprescribing, actions will be counted via a comparison of EMR-extracted medication lists from the 90 days before and after study enrollment. Charts will be reviewed for clinician notation on whether deprescribing (discontinuation or nonrenewal) was due to a cognitive indication [92]. We will consider the medications listed under “Drugs with strong anticholinergic properties” in the American Geriatrics Society Beers Criteria [93].

All primary and secondary outcomes are collected prospectively from the EMR. Textbox 2 presents a summary of the primary and secondary outcomes.

**Study outcomes.**

**Primary outcome: new cognitive diagnosis (with associated International Classification of Diseases, Tenth Revision, code [94])**

- Vascular dementia with or without modifiers (F01)
- Dementia in other diseases with or without modifiers (F02)
- Unspecified dementia with or without modifiers (F03)
- Mild cognitive impairment or minor neurocognitive disorder (unless patient entered study already having this diagnosis; G31.84)
- Alzheimer’s disease with late onset (G30.1)
- Other Alzheimer’s disease (G30.8)
- Alzheimer’s disease, unspecified (G30.9)
- Frontotemporal dementia (G31.09)
- Lewy body dementia (G31.83)
- Dementia associated with alcoholism (F10.27)
- Unspecified with psychoactive substance–induced persistent dementia (F19.97)
- Variant Creutzfeldt-Jakob disease (A81.01)
- Creutzfeldt-Jakob disease, unspecified (A81.00)
- Other Creutzfeldt-Jakob disease (A81.09)

**Composite secondary outcome: “improved dementia care” through relevant actions**

- Medications: donepezil, rivastigmine, galantamine, memantine, donepezil-memantine, antiamyloid monoclonal antibody infusions, and any other Food and Drug Administration–approved medications for dementia
- Deprescribing anticholinergic medications: discontinuation or nonrenewal of any of the 41 medications in table 7 (“Drugs with strong anticholinergic properties”) of the American Geriatrics Society Beers Criteria [93]
- Laboratory tests (cognitive indication only): thyroid-stimulating hormone, vitamin B12, glycated hemoglobin, complete blood count, basic metabolic panel, liver tests, HIV antigen and antibody combination, and syphilis immunoglobulin G and immunoglobulin M antibody with reflex
- Imaging tests (cognitive indication only): computerized tomography (head without contrast) and magnetic resonance imaging (brain without contrast)
- Specialist referrals for cognitive evaluation: geriatrics, neurology, neuropsychology, psychiatry, geriatric psychiatry, and social work
- Community referrals: Alzheimer’s Association or local support organization

#### Implementation Outcomes

**Figure 6:**
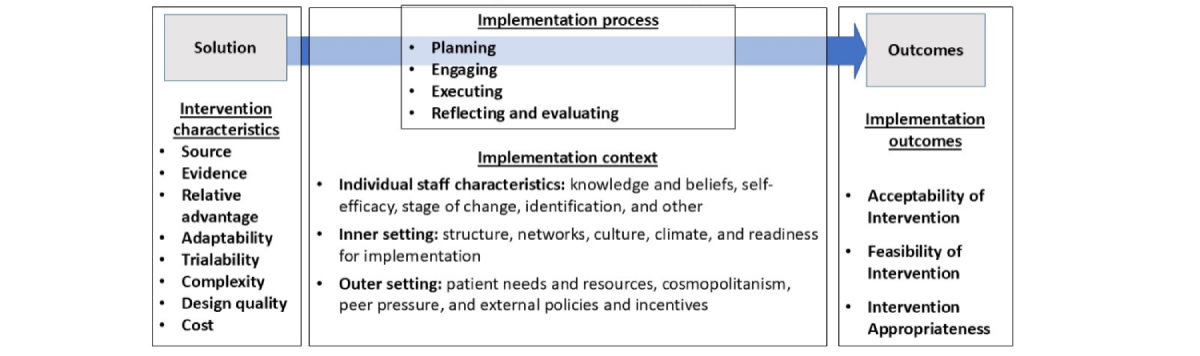
Consolidated Framework for Implementation Research (CFIR) Measurement Model.

We will begin recruiting implementation study participants after approximately 1 month of patient involvement at each study site, once a “steady state” has been established. We will vary time points for the collection of implementation outcomes throughout our engagement at each clinic in anticipation that informant responses may shift with varied exposure to the 5-Cog. Surveys will assess the implementation outcomes of acceptance, feasibility, and appropriateness, which are considered “leading indicators” of implementation success. These will be measured using the AIM, FIM, and IAM, as noted in the Interventions for the Implementation Study subsection [62,95]. The results of the surveys will be computed using descriptive statistics (central tendency and variability) and, after checking for assumptions (eg, high interclass intraclass correlation), aggregated at the clinic level across all respondents. We will also collect and analyze EMR engagement data (eg, the rates and types of responses to alerts and the use of order sets) to assess the acceptance of EMR tools. In addition, we will collect field notes with observations about the study sites and observations from informal conversations with clinic staff during the study period to better understand the implementation context. Figure 6 summarizes the implementation constructs and the CFIR outcome measurement model for the study.

#### Cost-Effectiveness Outcomes

The cost calculator includes parametric models of cost inputs; cost model outcomes from this study will thus allow scenario analyses [67,84]. The proposed calculator will incorporate both the direct costs of the intervention (test administration, staffing costs, etc) and costs associated with interventions prompted by the 5-Cog (brain imaging, specialist referral, laboratory tests, etc). The cost calculator will thus enable others to determine 5-Cog delivery costs and implementation specifics such as the cost of hiring at future implementation sites. Data collected for the BIA will determine financial planning and affordability, while data collected for the CEA will determine the effectiveness and value of the 5-Cog paradigm compared to enhanced usual care.

#### Exploratory Outcomes

Medical billing for cognitive impairment detection paradigms may be an important component of sustainability [22] because PCPs often lack sufficient resources to support the effort required to care for patients with complex needs [96]. The 5-Cog may have benefit in enhancing billing opportunities because it incorporates 5 minutes of cognitive testing and may add to the level of medical complexity of a visit. As noted, one of the 5-Cog 2.0 study interventions—implemented at both 5-Cog and control sites—is provider education on billing for cognitive impairment detection, diagnosis, and treatment. Although the 5-Cog study does not include PCP billing interventions as part of its aims, we will monitor and compare the rates of use of “cognitive” billing codes (eg, Medicare AWV and current procedural terminology codes 96136 and 96138 [97]) between the 5-Cog and enhanced usual care sites. At the 5-Cog sites, we will examine whether the use of “cognitive” billing codes is associated with 5-Cog paradigm administration.

### Assignment of Interventions

#### Sequence Generation

As in other pragmatic cluster randomized trials [98], we randomize at the clinic level. Commitment to join the study was sought from clinics. Once an adequate number of clinics (n=22) were identified, they were randomized. Computer-generated randomization assigned primary care practices in a 1:1 ratio within each site (the Bronx and Indiana) to either the 5-Cog paradigm or control arm. Randomization was stratified within each arm by clinic size (number of PCPs).

#### Allocation Concealment Mechanism

Randomization is central, computer generated, and not vulnerable to researcher influence. The study statisticians are blinded to interventions and other aspects of the trial.

#### Implementation

The 22 clinic clusters will be phased in gradually for logistical reasons, following a standardized procedure at each clinic: an overview presentation for leadership, a readiness presentation for providers and staff, and then study initiation.

#### Masking

Blinding of patients or PCPs at 5-Cog sites will not be feasible due to the nature of the intervention. However, the outcome is EMR based, and data abstractors will be blinded to allocation. Standardized checklists will be used for outcome measurement to further minimize bias. Statisticians will not be involved in the intervention delivery.

### Data Collection, Management, and Analysis

#### Data Collection Methods

Data will be collected from 2 sources (refer to the following subsections).

##### EMR Data

We will collect EMR data on trial-enrolled patients aged ≥65 years receiving care in the participating practices after obtaining consent from the practice leadership and receiving a waiver from the institutional review board (IRB). Outcomes will be extracted and aggregated from the EMR. Deidentified analytic databases will be created and analyzed by investigators.

##### Feedback From Care Providers, Staff, and Patients

Data on clinician and practice staff perceptions of the intervention and factors that facilitated or impeded implementation and sustainability will be obtained through digital surveys and in-person interviews with key informants. Data on patient perceptions and experiences with the 5-Cog will be obtained through in-person, telephone, and video call platform interviews. Data collection will be the responsibility of the clinical trial staff at the site under the supervision of the site investigators. The investigator will be responsible for ensuring the accuracy, completeness, legibility, and timeliness of the data reported. All source documents will be completed in a neat, legible manner to ensure accurate interpretation of data. Hard copies of the study visit worksheets will be provided for use as source document worksheets for recording data for each participant consented and enrolled in the study. Data recorded in the electronic case report form derived from the source documents will be consistent with the data recorded on the source documents. All effectiveness and implementation data will be entered into REDCap (Research Electronic Data Capture; version 14.0.31; Vanderbilt University), a 21 CFR Part 11–compliant data capture system provided by the Albert Einstein College of Medicine through an NIH grant (UM1TR004400). The data system includes password protection and internal quality checks, such as automatic range checks, to identify data that seem inconsistent, incomplete, or inaccurate.

#### Data Management

Data collected for this study will be analyzed and stored at the Albert Einstein College of Medicine via REDCap. These data will be available through web-based registration to researchers for future analyses after the completion of our pragmatic clinical trial and publication of the main results. For resource-sharing approval, collaborators—both internal and external—will be required to register on the web and submit a resource transfer agreement. Requests can be submitted electronically to expedite research progress. Requests will also be reviewed by the steering committee at regularly scheduled meetings.

#### Effectiveness Study: Statistical Plan

The baseline characteristics of the clinical sites as well as individual enrolled patients will be examined after the baseline measurement time point at the halfway point of target enrollment. It is possible that baseline differences between the groups or missing data will limit the value of data analysis of measurements. Effects on the power to detect differences in the primary outcome will be evaluated and communicated to the principal investigators (PIs), data safety monitoring board (DSMB), and NIH. Given the monitoring plans outlined in the Monitoring subsection, it is exceedingly unlikely that there will be baseline differences between the groups of a magnitude that could threaten the validity of the study.

We will evaluate the clinical effectiveness of the 5-Cog paradigm on new cognitive impairment detection by comparing the proportion of “new MCI and dementia diagnoses” by PCPs within 90 days after the clinic encounter in the 5-Cog paradigm group versus the enhanced usual care group using generalized linear mixed effects (GLME) models with a logit link to account for clinic-clustered data [99]. Using these models, odds ratios of new MCI and dementia diagnoses between the 5-Cog paradigm and enhanced usual care arms will be calculated and tested. We chose a 90-day cutoff because the majority of PCP actions occurred within the first 30 days in our recent RCT [33]. The 22 clinics are cluster randomized to either the 5-Cog paradigm or enhanced usual care arm, which can result in correlated data due to clustering of patients within clinics. This issue will be handled using random effects in the mixed models. Proportions of new MCI and dementia diagnoses for each treatment group will also be reported. We will perform an intention-to-treat analysis. As a secondary approach, we will also perform a per-protocol analysis. All estimates will be reported together with their 95% CIs. The same method will be used for the secondary outcome (“improved dementia care”).

Stratified analyses across the 22 sites will be performed for groups impacted by health disparities, as designated by the NIH, including those from racial and ethnic minority groups (Black and Hispanic individuals) and individuals from socioeconomically disadvantaged backgrounds. Interactions between race and ethnicity and socioeconomic status (SES) will also be explored by stratified analyses by the combination of the 2 factors and by including the 2 factors and their product as well as interactions with the treatment groups in the GLME model for all enrolled patients. We will compare 5-Cog results by location in sensitivity analyses to detect any health care system–based variations.

All sample size and power calculations were performed using 2-tailed tests with a significance level of α=.05. The within-cluster intraclass correlation in this cluster randomized trial is assumed to be 0.05 based on prior studies [100]. On the basis of our RCT data, the proportions of “new MCI and dementia diagnoses” are expected to be 14% and 2% among the 5-Cog and control arms, respectively. The proportions of “improved dementia care” are expected to be 21% and 7% among the 5-Cog and control arms, respectively. In our total sample (N=6600), we have >99% power to detect the overall effectiveness of the 5-Cog paradigm on “new MCI and dementia diagnoses” and the secondary outcome of “improved dementia care.” Sample size determination was based on the primary outcome among individuals from racial and ethnic minority groups (2112/6600, 32%) and those with low SES (3750/6600, 56.82%). Assuming that 80% (1400/1800) of the participants in the 6 Bronx clinics are from racial and ethnic minority groups, and 75% (1350/1800) have low SES, as well as 14% (672/4800) and 50% (2400/4800), respectively, in the 16 Indiana clinics, 300 patients per cluster (clinic) will enable the detection of the effect of the 5-Cog paradigm on new MCI and dementia diagnoses with 81% power among individuals from racial and ethnic minority groups and 99% power among those with low SES.

Our outcomes are ascertained from the EMR by blinded assessors and do not require repeat in-person assessments. This will minimize missing data issues. We will implement robust EMR-based data management processes to further minimize missing data. Cluster sites are not contiguous, and patients within a cluster are assigned to specific PCPs who do not work across sites. Any intentional or unintentional contamination will be monitored by the data team, including by comparing enrolled patient lists across the sites.

#### Implementation Study: Statistical Plan

Interview data will undergo content analysis to identify and score implementation barriers or facilitators. Qualitative content analysis will be performed primarily using a deductive approach, starting from a developed theoretical model—the CFIR—and its associated hypotheses and then examining data to disconfirm or confirm these hypotheses [101]. The analysis will also allow for the inductive development of new themes, categories, and relationships as they arise from the data [102]. Deductive data analysis will be guided by the CFIR constructs and their definitions such that units of qualitative data (sentences or passages) are assigned to ≥1 codes reflecting the CFIR constructs [102]. We will use NVivo 15 (Lumivero) to manage and code qualitative data. For each implementation interview, there will be an artificial intelligence–generated transcript that will be manually reviewed by study staff before it is moved into NVivo. To guide systematic coding, a codebook will be developed. A multiple-analyst team will be trained and tested on its application. Analysts will code a random subset of interviews together during training and then assigned at a ratio of 2 to 3 per transcript for either all or a subset of transcripts, depending on the original level of coding agreement (ie, interrater reliability) [103,104]. Subsequent coding discussions will be held intermittently for the calibration and discussion of difficult cases and disagreements. Interrater reliability will be computed in NVivo at the start, middle, and end of coding, with retraining and adjustments to the plan depending on the results. Inductively, we will allow for the emergence of new codes and subcodes [104]. We will also use applied thematic analysis [105] to identify patterns within and between codes. As much as possible, data analysis will occur in parallel with data collection, enabling us to adjust data collection procedures to capture emerging and unexpected phenomena, as well as perform member checking with informants willing to complete follow-up interviews to review and validate our coding of their initial interviews. Within each CFIR code, for each implementation context or process element, the assigned analyst will add a rating on a scale ranging from +3 (strong facilitator) to −3 (strong barrier) [54]. Analysts will be trained to use evidence from interviews and not personal opinions to rate each driver [102].

RAs in Indiana and the Bronx will be trained by coinvestigators to reliably collect implementation process data from a combination of documents, field notes, and interviews, with select staff responsible for the implementation process. To capture implementation data monthly, while minimizing staff burden, we will use the Prospectively Reported Implementation Update and Score [106]. The analysts will reconstruct these data into process maps or workflow diagrams depicting key historic moments, process variations, and players [54,59,102].

#### Linking Implementation to Effectiveness Analysis

Examining how stakeholder-level factors impact and are impacted by the 5-Cog implementation is critical because implementation can be influenced by stakeholders’ characteristics, attitudes, intentions, and motivations. Stakeholder-level factors are also shaped by organizational factors [107]. Following the guidance of Damschroder and Hagedorn [58], we will link identified and rated (+3 to −3) context and implementation process drivers to implementation outcomes. Our analytical approach is as follows: we will derive clinic-level mean and variability measures of the AIM, FIM, and IAM scales and examine whether these implementation measures are associated with cognitive impairment detection by using GLME models with a logit link, treating the mean and variability measures of the AIM, FIM, or IAM as independent variables [108]. This analysis will determine how detection rates are associated with the levels or variabilities in implementation acceptance, perceived feasibility, and appropriateness within each clinic. We will explore whether and how context variables account for 5-Cog effects on outcomes by including quantitative context measures and interactions with the intervention group as independent variables in the GLME models from the effectiveness analysis [108]. We will then develop a thematic matrix that includes characteristics derived from stakeholder surveys and emerging themes from our qualitative data for each 5-Cog clinic site and conduct side-by-side comparisons—at system, organizational, and staff levels—of the factors that were identified as facilitating or hindering 5-Cog implementation. We will note common and unique factors for each clinic or location. This will generate a list of relevant system-, organizational-, and staff-level factors and processes organized by level of consensus (ie, identified by >1 source) and operational salience (ie, identified as critical for implementation). We will use this list to generate a heuristic model to inform future implementation strategies [54,55].

#### CEA and Economic Impact

Using data from the BIA cost-tracking tool, the cost-effectiveness ratio [109,110] “numerator” will account for site personnel and other costs at both intervention and control sites. The denominator will represent the number of cognitive impairment cases identified at the 5-Cog and control sites. In summary, the cost-effectiveness ratio represents the incremental cost of the 5-Cog intervention compared to enhanced usual care to achieve a clinically meaningful change, divided by the difference in effectiveness, to determine the cost per cognitive impairment case identified. This is expressed as follows:

Cost-effectiveness ratio = intervention costs + Δ (health care costs) / Δ health outcomes (5-Cog) – Δ health outcomes (enhanced usual care comparison group)

To assess uncertainty in our analysis, we will follow the recommendations of the International Society for Pharmacoeconomics and Outcomes Research and the Society for Medical Decision Making Modeling Good Research Practices Task Force [63,65-68,111]. Variability of inputs across study sites with differing levels of social determinants of health will be included in analyses [112]. Differences in intervention and health care costs due to secondary procedures will be assessed individually using negative binomial regression and reported with a 95% CI. Only statistically significant differences from the regression analyses will be incorporated into the cost-effectiveness ratio. Differences in health outcomes will be assessed as described in the Effectiveness Study: Statistical Plan subsection, and the estimate of the GLME model, if significant, will be incorporated into the cost-effectiveness ratio. Costs will be discounted at a 3% annual rate for the trial period, as recommended by the Second Panel on Cost-Effectiveness in Health and Medicine [113].

### Monitoring

#### Data Monitoring

Each clinical site will perform internal quality management of study conduct, data collection, documentation, and completion. All sites will follow a common quality management plan. Before we began data collection, the PIs and the DSMB chair reconfirmed that our sites have appropriate safety measures in place. The DSMB met with the entire research team to review the study protocols. Particular attention was paid to outcome definition, study design, procedures for recording and reporting adverse events (AEs), informed consent procedures, and documentation. At its initial meeting, the DSMB approved the protocol and formulated its operating procedures (eg, meeting schedule, report deadlines for the study statistician, unblinding policy, and guidelines for releasing interim data to the investigators). At this meeting, the plans for interim monitoring for efficacy and futility were presented to the DSMB to aid in trial oversight. We will train competent staff to conduct the assessments and ensure that they understand the data collection procedures and process as well as AE reporting requirements.

#### Effectiveness Study: Monitoring for Harms

Patients who receive care at the practices randomized to implement the 5-Cog paradigm may experience minimal risk through their participation. It is possible that patients may feel shame, anxiety, and some emotional discomfort when completing the cognitive assessment; however, this may be the case with the standard of care cognitive assessment as well and may not be specific to the intervention arm. In addition, in our ADRD screening trial that evaluated the potential benefits and harms of ADRD screening, there were no differences between the screened and control groups in quality of life, depressive symptoms, or anxiety [114,115]. That trial also assessed the impact of this early diagnosis on participants’ quality of life and care and found no negative impact on quality of life, depression, anxiety, health care use, or independence at home [114,115]. The risk of potential harm such as stigma is not known. However, the early detection of ADRD is considered part of the standard of care, and screening for ADRD is part of the AWV covered by Medicare because of the significant potential benefits.

#### Implementation Study: Monitoring for Harms

Answering questions on surveys and participation in interviews with trained personnel involve minimal psychological, social, or other risks. We do not expect any AEs during these noninvasive assessments.

#### Full Study: Monitoring for Harms

Safety oversight will be under the direction of a DSMB composed of individuals with the appropriate expertise. We will set up a data safety monitoring plan, which will be monitored by the PIs and DSMB. The PIs will also conduct data and safety monitoring and will regularly monitor study progress, goal achievement, and overall research direction in consultation with the coinvestigators. Members of the DSMB will be independent from the study conduct and free of conflicts of interest. The DSMB, which will meet at least semiannually to assess safety and efficacy data from each study arm, will operate under the rules of an approved charter that was written and reviewed at its organizational meeting. The DSMB will provide its input to the National Institute of Neurological Disorders and Stroke and NIH staff.

AEs will be monitored on an ongoing basis by the study managers through self-initiated reports from participants or patients, observations of research and clinic staff at the intervention sites, biannual review of acute care use among enrolled patients, and review of EMR data at 90-day outcome collection for any medical records or notes of medical, psychological, or stress symptoms possibly related to the administration of the 5-Cog battery. In addition, a list of hospitalizations and emergency department visits (for any reason) occurring within 90 days after the clinic visit will be generated by the EMR system, which will be reviewed by the PIs and study investigators. All AEs not otherwise precluded per the protocol will be captured on the appropriate case report form. Information to be collected includes event description, time of onset, clinician’s assessment of severity, relationship to study procedures (assessed only by those with the training and authority to make a diagnosis), and time of resolution or stabilization. All AEs occurring during the study will be documented appropriately regardless of their relationship to the intervention and followed until adequately resolved. Any preexisting medical or psychiatric conditions at screening will be considered baseline and not reported as an AE. However, any deterioration in a patient’s clinical or psychological condition during the assessment will be recorded as an AE. Changes in AE severity will be documented to assess the duration of the event at each severity level. For AEs characterized as intermittent, the onset and duration of each episode will be documented. New diagnoses of MCI or dementia may result in psychological stress for patients that may not be captured in the EMR. Hence, we will also assess these more subtle psychological symptoms during the implementation interviews.

### Auditing

Quality control procedures will be implemented. In the implementation study only, regarding informed consent, study staff will review both the documentation of the consenting process and a percentage of the completed consent documents to evaluate compliance with good clinical practice, accuracy, and completeness. Feedback will be provided to the study team to ensure that proper consenting procedures are followed. To assess data accuracy (the level of correctness of stored information [116]), site staff will compare a representative sample of source data against the database, focusing on key data points. The study team will review protocol deviations on an ongoing basis and implement corrective actions if deviations reach a concerning level. Should independent monitoring become necessary, the PIs will provide direct access to all trial-related sites, source data and documents, and reports for monitoring and auditing by the funding agency and inspection by local and regulatory authorities.

### Ethical Considerations

The Albert Einstein College of Medicine IRB approved this study (2022-14144) and serves as the IRB of record for both sites under a reliance agreement with the Indiana University IRB. This study will be conducted in accordance with federal publication and data-sharing policies and regulations. Regarding consent for participants, we received a waiver of informed consent from individual patients from the IRB to maintain the pragmatic design of the clinical trial. For the implementation part of the study, consent is obtained from all participants before completion of any study measures. Participants who consent for the implementation study receive US $25 in compensation for completed interviews. Outcomes will be extracted and aggregated from the EMR and will be deidentified. After the study is completed, the deidentified, archived data will be transmitted to and stored in a data repository with protections in place for patient confidentiality.

## Results

The grant was funded, and the protocol received IRB approval, in September 2022.

### Effectiveness Study

A validation cohort of 76 older patients (mean age 76.5, SD 7.72 y; n=50, 66% women) from dementia centers at Montefiore Einstein was evaluated with the 5-Cog battery to determine optimal cut scores. This was completed by July 2023. Among these 76 patients evaluated by the multidisciplinary care provider team at these centers (neuropsychology, neurology, and geriatrics), the resulting clinical diagnoses were as follows: 32 (42%) had a diagnosis of dementia, 28 (37%) MCI, 12 (16%) subjective cognitive concerns without objective cognitive impairment, and 2 (3%) had no cognitive diagnosis assigned. The mean scores on the PMIS were 5.8 (SD 2.7), Symbol Match 27.2 (SD 18.2), and sMCR 2.4 (SD 1.7). We correlated performance on the individual 5-Cog battery tests with the Blessed Information-Memory-Concentration Test scores (range 0-32; higher scores indicate worse performance), an omnibus test of general mental status (mean 10.3, SD 6.8). The PMIS (Pearson *r*=−0.76; *P*<.001) and Symbol Match (*r*=−0.69; *P*<.001) showed excellent correlation with Blessed scores. While the sMCR was not correlated with Blessed scores (*r*=0.008; *P*=.95), it was correlated (*r*=−0.34; *P*=.007) with walking speed (mean 72.3, SD 25.3 cm/s) in this sample. The PMIS was correlated (*r*=−0.59; *P*<.001) with the 5-item memory recall subtest on the Blessed Information-Memory-Concentration Test (mean 2.9, SD 1.84; higher scores indicate worse performance). Symbol Match was correlated (*r*=0.88; *P*<.001) with the Symbol Digit Modalities Task score (mean 21.2, SD 14.6; lower scores indicate worse performance).

As we examined the sensitivity and specificity data to choose cut scores, we chose to favor sensitivity to minimize missing individuals with true disease in this sample of patients considered high risk because of their cognitive concerns. The cut scores for a *positive* result on the 5-Cog components were as follows: PMIS ≤6 (range 0-8), Symbol Match ≤25 (range 0-65), and sMCR >5 (range 0-7). There was a high rate of completion of the PMIS (75/76, 99%), Symbol Match (76/76, 100%), and sMCR (74/76, 97%) tests on the 5-Cog battery, indicating high acceptability and feasibility of administration of the 5-Cog battery in populations with cognitive impairments.

The DSMB met in July 2023, approved the protocol and data safety monitoring plan, and authorized study enrollment. By October 2023, all 22 clinics had agreed to participate and been randomized. By November 2023, decision support tool integration into the EMRs had been finalized. As of December 2024, a total of 12 clinics had completed onboarding processes (refer to the Methods section, Care Provider Education and Preparation subsection), and 2369 patients had been enrolled.

### Implementation Study

We have completed 6 implementation interviews and 23 implementation surveys over 12 clinic sites.

### Cost-Effectiveness Study

5-Cog 2.0 investigators have already collected procedure and health care cost estimate data for Montefiore-Einstein for a 5-Cog 1.0 CEA. Preliminary results suggest that the 5-Cog paradigm is a cost-effective option compared to “enhanced usual care” for the early detection of cognitive impairment in primary care (H Congivaram, BS, unpublished data, December 2024). Investigators have begun collecting cost estimates for 5-Cog procedures and resulting health care costs in the IUH system, as these were not previously collected.

## Discussion

### Anticipated Findings

The results of this 5-Cog 2.0 RCT will provide additional evidence that the 5-Cog paradigm is effective in improving dementia diagnosis. At the same time, the implementation portion of this study will provide critical information about the context, facilitators, and barriers to using the 5-Cog successfully, while the cost-effectiveness portion of this study will offer a practical analysis of the cost implications of the paradigm. Taken together, these study outcomes will allow informed, tailored, and rapid adaptation and dissemination of the 5-Cog paradigm to diverse primary care environments.

Meeting the ongoing and anticipated growing need for timely dementia diagnosis and care has been a focus of much study. Despite the availability of high-quality tools for dementia detection [22], significant gaps remain in real-world application, and the rates and timeliness of diagnosis remain unacceptably low [28,117,118]. This translates to clinical “blind spots” for care providers and missed opportunities for patients. These gaps are even more pronounced among individuals from historically minoritized racial and ethnic groups [5,119]. The 5-Cog paradigm is poised to effectively bridge these gaps. The 5-Cog itself is designed for widespread applicability, with characteristics that make it appropriate for individuals from various cultural, linguistic, and literacy backgrounds [32,33]. The 5-Cog’s simple, fast format also makes it easily adaptable in resource-limited environments in contrast to some cognitive assessment tools that require specialized training or equipment to administer [120,121]. Although the evidence for decision support systems to improve clinical practice is mixed [50,122], the 5-Cog paradigm does build on prior evidence of the efficacy of decision supports in dementia detection [123,124].

### Strengths and Limitations

This study has several strengths. Its results are anticipated to have improved generalizability compared to prior studies for several reasons: its large sample size; the racially and ethnically diverse patient population being enrolled; and the pragmatic nature (using local CHW staff, colocation in patients’ usual sites of care, and a waiver of written informed consent), which may help reduce the hesitancy of individuals from historically marginalized groups to participate in the trial [125]. While the characteristics and specifications of primary care clinic sites will vary, the urban and rural primary care clinic sites in the 5-Cog study are typical of primary care clinics in the United States, especially those that serve underserved individuals from historically minoritized groups. Prior studies of decision supports in dementia were limited by a small sample size and a lack of active control groups [50,122]. The 5-Cog 2.0 study overcomes these limitations. Another strength of this study is that the BIA will form the basis of a tool that can be used to tailor financial adoption and scalability in different practice settings for national implementation. Finally, this study’s unique hybrid effectiveness-implementation design will rapidly generate actionable data to advance progress in closing dementia care gaps. This study is anticipated to have a few limitations. Although the inclusion criteria are intentionally liberal in this pragmatic trial, the trial may not address dementia care gaps for the subset of patients who lack insight into their cognitive impairments and deny having them and are therefore not eligible for the trial. Implementation analyses will allow an exploration of whether and how the 5-Cog can support PCPs’ clinical approach to this subset of patients who do not recognize their own cognitive decline. A limitation of the CEA is that the time horizon is relatively short, and long-term sustainability will need to be assessed in future studies.

### Anticipated Dissemination Strategy

The information gained about the 5-Cog paradigm, including details of the EMR-embedded clinical decision support and incorporation into medical billing systems, in this pragmatic trial will enable future implementation in everyday clinical settings across the United States for routinely detecting cognitive impairment, including dementia. For dissemination, this trial has been registered at ClinicalTrials.gov (NCT05515224), and results information from this trial will be submitted to ClinicalTrials.gov. Every attempt will be made to publish the results in peer-reviewed journals. Final research data will be shared openly and in a timely manner: data from this study may be requested from other researchers 1 year after the completion of the primary end point by contacting Albert Einstein College of Medicine. For dissemination of the 5-Cog paradigm itself, the 5-Cog forms and administration instructions will be made available on a publicly facing website. 5-Cog team members anticipate making presentations at national conferences of clinical, educational, and administrative leaders in dementia care to facilitate the dissemination of the paradigm.

### Future Directions

Although there will not be a “one-size-fits-all” solution to detecting cognitive impairment across the country, we anticipate that elements of the 5-Cog battery can be adapted in distinct sites and further studied; for instance, pictures in the PMIS may need to be updated to account for local cultural and ethnic factors. We also anticipate that, as new knowledge regarding MCI and dementia and treatments emerges in the future, we will have to update the 5-Cog decision tree and adapt to other EMR systems. Finally, the results of this study will provide the foundation for further study of the 5-Cog assessment for use in the increasingly common collaborative dementia care models [15,30,126-128], as a screening paradigm for population health models [129,130], and to evaluate the impact of early detection on clinical care and outcomes.

## Abbreviations

ADRD: Alzheimer disease and related dementias
AE: adverse event
AIM: Acceptability of Intervention Measure
AWV: annual wellness visit
BIA: budget impact analysis
BPA: best practice advisory
CEA: cost-effectiveness analysis
CFIR: Consolidated Framework for Implementation Research
CHW: community health worker
DSMB: data safety monitoring board
EMR: electronic medical record
FIM: Feasibility of Intervention Measure
GLME: generalized linear mixed effects
IAM: Intervention Appropriateness Measure
IRB: institutional review board
IUH: Indiana University Health
MCI: mild cognitive impairment
MCR: motoric cognitive risk
NIH: National Institutes of Health
PCP: primary care provider
PMIS: Picture-Based Memory Impairment Screen
RA: research assistant
RCT: randomized controlled trial
REDCap: Research Electronic Data Capture
SCQ: subjective cognitive concern questionnaire
SES: socioeconomic status
sMCR: Subjective Motoric Cognitive Risk

## Data Availability

The data that support the findings of this study will not be publicly available due to reasons of confidentiality. On reasonable request to the corresponding author, individual deidentified participant data (including data dictionaries) will be made available via a REDCap (Research Electronic Data Capture) web-based database, after review and approval of a methodologically sound proposal, with a signed data access agreement, in line with ethics committee requirements.
